# High Neutrophil-To-Lymphocyte Ratio (NLR) and Systemic Immune-Inflammation Index (SII) Are Markers of Longer Survival After Metastasectomy of Patients With Liver-Only Metastasis of Rectal Cancer

**DOI:** 10.3389/pore.2022.1610315

**Published:** 2022-04-27

**Authors:** Nándor Polk, Barna Budai, Erika Hitre, Attila Patócs, Tamás Mersich

**Affiliations:** ^1^ Department of Laboratory Medicine, Semmelweis University, Budapest, Hungary; ^2^ Departmet of Visceral Surgery, National Institute of Oncology, Budapest, Hungary; ^3^ Department of Molecular Genetics, National Institute of Oncology, Budapest, Hungary; ^4^ Medical Oncology and Clinical Pharmacology “B” Department, National Institute of Oncology, Budapest, Hungary; ^5^ Clinical Central Laboratory, National Institute of Oncology, Budapest, Hungary

**Keywords:** neutrophil-to-lymphocyte ratio, liver-only metastases of rectal cancer, metastasectomy, relapse-free survival, systemic immune-inflammation index, liver-only metastases of colon cancer, preoperative treatment

## Abstract

**Background:** The literature data regarding colon cancer patients with liver-only metastases (CLM) show that NLR determined before metastasectomy is a prognostic marker of shorter relapse-free survival (RFS), but no results has been reported to date for rectal cancer patients with liver-only metastases (RLM). This study aimed to investigate the NLR and SII in CLM and RLM.

**Methods:** Relapse-free (RFS) and overall survival (OS) were evaluated in 67 CLM and 103 RLM patients with a median follow-up of 46.5 and 59.8 months, respectively. Pre- and/or postoperative chemotherapy ± targeted treatment was applied in 96% and 87% of CLM and RLM patients, respectively. The cut-off level for hematologic parameters were determined by receiver operating characteristic (ROC) analysis. Univariate analysis was performed by Kaplan-Meier method and log rank test. For multivariate analysis Cox regression was applied.

**Results:** In univariate analysis low NLR (cut-off 2) and SII (535) were predictors of longer RFS in case of CLM (*p* < 0.01). In contrast, for RLM high NLR (2.42) and SII (792) were predictors of longer RFS (*p* < 0.001). For RLM both NLR and SII proved to be independent markers of RFS (HR 0.66 (95% CI 0.52–0.84) and 0.73 (0.57–0.91), respectively) and OS (0.76 (0.58–0.99) and 0.66 (0.5–0.87), respectively). Only NLR (1.44 (1.04–1.99)) was independent marker of RFS for CLM. The preoperative treatment has not influenced the role of NLR or SII.

**Conclusion:** In contrast to CLM, in RLM the high NLR or SII determined before metastasectomy proved to be independent prognostic factors of longer RFS and OS.

## Background

In Hungary the incidence and mortality of colorectal cancer (CRC) is currently the second most common among malignancies [1]. Liver metastases develop in nearly half of CRC patients, and the best treatment for patients with colorectal liver-only metastases (CRLM) is the surgical resection, however, 60–80% of them experience recurrence after resection [2]. Knowledge of derived preoperative hematologic parameters, which are readily available data, may be important in assessing the risk of recurrence. The best known prognostic parameter is the neutrophil-to-lymphocyte ratio (NLR), which has also been studied in several studies evaluating patients with CRLM [3, 4, 5]. In these studies, patients were dichotomized based on a calculated or from the literature taken cut-off value, and based on this, significantly different relapse-free survival (RFS) curves were found. In general, the lower NLR has been identified as a marker of longer RFS, but at the same cut-off value (e.g., Ref. 5), strongly significant [6, 7] or non-significant [8, 9] differences of RFS curves were demonstrated. All these data together reflect the incoherent association. The systemic immune-inflammation index (SII = platelet count x NLR), which has been shown to be the best prognostic marker for occurrence of liver metastasis in CRC [10], was investigated in only two studies for the recurrence in CRLM [11, 12].

In previous studies, the presence of pre- and postoperative chemo- and targeted therapies was not an exclusion criterion, although in some studies all patients received pseudoneoadjuvant (hereafter preoperative) [13, 14, 15] or pseudoadjuvant (hereafter postoperative) treatment [16, 17]. The difference in rate of pre- and postoperative treatments reflects data from everyday practice. The localization of the primary tumor has not been detailed in many studies, and the calculation method of cut-off values is also not uniform; the most common is based on ROC analysis. Several studies exclusively investigated the overall survival (OS), but their results are also non convergent [18, 19, 20].

Based on our previous experience [21] and moreover on the histologic, genetic, behavioral, etc. differences between colon and rectum tumors detailed by Paschke et al. [22] and [23, 24], we hypothesized that the role of NLR may depend on the site of primary tumor of CRLM patients. For colon cancer Chang et al. [25] proved that low NLR is a significant marker of RFS, but no report was found for rectal cancer. The aim of the present study was to separately investigate the colon and rectal cancer patients with liver-only metastases wether NLR and SII determined before metastasectomy are possible markers of RFS and OS.

## Materials and Methods

### Patients

All patients underwent curative resection of primary cancer. Those patients whom liver metastases were surgically treated between 2001 and 2018 were reviewed (*n* = 205). The exclusion criteria were: 1) Radio-frequency thermal ablation (RFTA) or radiofrequency ablation (RFA) of liver metastases (*n* = 16); 2) unavailable laboratory parameters (*n* = 16); 3) presence of other synchronous malignancies (*n* = 3). A total of 170 patients were included in the study, 67 of whom had a primary tumor in the colon (CLM) and 103 in the rectum (RLM). Besides the clinicopathologic parameters the presence of chemotherapy (±targeted treatment) before and/or after metastasectomy was recorded. The 5-FU-based chemotherapy was administered alone or combined with oxaliplatin or irinotecan. Targeted therapy (cetuximab, bevacizumab or panitumumab) was also applied in several cases. All hematological parameters were determined from the blood samples taken before metastasectomy.

Metastasectomy was laparoscopic (16 and 18%, for CLM and RLM, respectively) or classic, including synchronous surgery of primary tumor and liver metastasis (10%, both CLM and RLM). In case of 10 RLM patients the “liver first” strategy was chosen. Hepatic magnetic resonance imaging (MRI) to assess the local disease extension and to evaluate chemotherapy response, thoraco-abdominal computed tomography (CT) and positron emission tomography (PET-CT) were systematically performed to evaluate the presence of disease. Follow-up of all patients was performed every 3 months (physical examination, abdominal ultrasonography, CT, MRI or PET-CT, and routine laboratory).

### Statistics

The primary objective was the prognostic value of NLR and SII for RFS; secondary objectives included OS and the effect of preoperative treatment on the role of NLR and SII. RFS was calculated from date of metastasectomy to date of progression or end of follow-up. OS was calculated from date of metastasectomy to date of cancer-related death or end of follow-up. The cut-off values for dichotomization of continuous variables were determined by ROC analysis of relapse or death for RFS and OS, respectively. The ratio of relapse was not underestimated because of enough follow-up duration. Survival curves were constructed by Kaplan-Meier method and compared by log rank test. Multivariate Cox regression analysis was used to find independent markers of survival. To avoid multicollinearity only uncorrelated variables were used in the Cox regression analysis. The NCSS program (NCSS 2019 Statistical Software (2019). NCSS, LLC. Kaysville, Utah, United States, ncss.com/software/ncss.) was used for statistical analyses.

## Results

The clinical and laboratory parameters of patients are presented in Table 1 and Table 2.

**TABLE 1:** Clinicopathological characteristics and preoperative laboratory parameters of patients with colon cancer liver-only metastases.

Parameters		N (%)	Median (range)	Cut-off value RFS/OS
Age (yrs)			65 (38–80)	62/66
	<62	23 (34)		
	≥62	44 (66)		
	<66	35 (52)		
	≥66	32 (48)		
Gender				
	male	36 (54)		
	female	31 (46)		
Type of surgery used for metastasectomy				
	laparoscopy	11 (16)		
	open	56 (84)		
Resection margin				
	R0	46 (69)		
	R1	21 (31)		
Synchronicity of primary surgery and metastasectomy				
	synchronous	7 (10)		
	metachronous	60 (90)		
Preoperative (metastasectomy) chemotherapy ± targeted therapy				
	none	16 (24)		
	yes	51 (76)		
	targeted	43 (84)		
Postoperative (metastasectomy) chemotherapy ± targeted therapy				
	none	18 (27)		
	yes	49 (73)		
	targeted	27 (55)		
Pre- or postoperative chemotherapy ± targeted therapy				
	none	3 (4)		
	yes	64 (96)		
	targeted	48 (75)		
WBC (G/l)			6.8 (3.5–12.5)	6.8/7.1
	<6.8	32 (48)		
	≥6.8	35 (52)		
	<7.1	35 (52)		
	≥7.1	32 (48)		
neutrophil (G/l)			4.3 (1.2–9.2)	4/4.8
	<4	27 (40)		
	≥4	40 (60)		
	<4.8	41 (61)		
	≥4.8	26 (39)		
lymphocyte (G/l)			2 (0.8–3.7)	1.94/2
	<1.94	32 (48)		
	≥1.94	35 (52)		
	<2	36 (54)		
	≥2	31 (46)		
platelet (G/l)			244 (105–446)	210/184
	<210	20 (30)		
	≥210	47 (70)		
	<184	10 (15)		
	≥184	57 (85)		
NLR			2 (0.7–8.8)	2/1.7
	<2	30 (45)		
	≥2	37 (55)		
	<1.7	18 (27)		
	>1.7	49 (73)		
SII (G/l)			502 (125–1952)	535/290
	<535	39 (58)		
	≥535	28 (42)		
	<290	9 (16)		
	≥290	58 (84)		
GOT (U/l)			25 (13–343)	24/24
	<24	22 (37)		
	≥24	37 (63)		
	NA	8		
GPT (U/l)			22 (9–296)	31/17
	<31	48 (80)		
	≥31	12 (20)		
	<17	14 (23)		
	≥17	46 (77)		
	NA	7		
Site of progression				
	liver	33 (63)		
	lung	5 (10)		
	liver+lung	7 (13)		
	other	7 (13)		
Extent of progression				
	single	41 (61)		
	multiple	11 (16)		
	none	15 (22)		

**TABLE 2:** Clinicopathological characteristics and preoperative laboratory parameters of patients with rectal cancer liver-only metastases.

Parameters		N (%)	Median (range)	Cut-off value RFS/OS
Age (yrs)			62 (31–81)	68/64
	<68	68 (66)		
	≥68	35 (34)		
	<64	58 (56)		
	≥64	45 (44)		
Gender				
	male	69 (67)		
	female	34 (33)		
Type of surgery used for metastasectomy				
	laparoscopy	19 (18)		
	open	84 (82)		
Resection margin				
	R0	65 (63)		
	R1	38 (37)		
Synchronicity of primary surgery and metastasectomy				
	synchronous	10 (10)		
	metachronous	93 (90)		
	“liver first”	10 (11)		
Preoperative (metastasectomy) chemotherapy ± targeted therapy				
	none	37 (36)		
	yes	66 (64)		
	targeted	41 (62)		
Postoperative (metastasectomy) chemotherapy ± targeted therapy				
	none	29 (28)		
	yes	74 (72)		
	targeted	23 (31)		
Pre- or postoperative chemotherapy ± targeted therapy				
	none	13 (13)		
	yes	90 (87)		
	targeted	49 (54)		
WBC (G/l)			5.9 (3.4–18.6)	7.3/4.2
	<7.3	79 (77)		
	≥7.3	24 (23)		
	<4.2	14 (14)		
	≥4.2	89 (86)		
neutrophil (G/l)			3.8 (2–13.5)	5.5/3.5
	<5.5	85 (83)		
	≥5.5	18 (17)		
	<3.5	37 (36)		
	≥3.5	66 (64)		
lymphocyte (G/l)			1.4 (0.4–3.6)	0.97/1.7
	<0.97	32 (31)		
	≥0.97	71 (69)		
	<1.7	77 (75)		
	≥1.7	26 (25)		
platelet (G/l)			210 (111–396)	161/314
	<161	18 (17)		
	≥161	85 (83)		
	<314	90 (87)		
	≥314	13 (13)		
NLR			2.9 (0.9–11.3)	2.42/2.56
	<2.42	31 (30)		
	≥2.42	72 (70)		
	<2.56	39 (38)		
	>2.56	64 (62)		
SII (G/l)			616 (189–3,500)	792/742
	<792	65 (63)		
	≥792	38 (37)		
	<742	63 (61)		
	≥742	40 (39)		
GOT (U/l)			23 (9–72)	25/20
	<25	50 (58)		
	≥25	36 (42)		
	<20	20 (23)		
	≥20	66 (77)		
	NA	17		
GPT (U/l)			19 (5–77)	22/13
	<22	52 (60)		
	≥22	34 (40)		
	<13	13 (15)		
	≥13	73 (85)		
	NA	17		
Site of progression				
	liver	43 (42)		
	lung	21 (20)		
	liver+lung	8 (8)		
	other	13 (13)		
Extent of progression				
	single	69 (67)		
	multiple	16 (16)		
	none	18 (17)		

GOT, aspartate aminotransferase; GPT, alanine aminotransferase; NA, not available; NLR, neutrophil-to lymphocyte ratio; OS, overall survival; RFS, relapse-free survival; SII, systemic immune-inflammation index; WBC, white blood cells.

The median follow-up was 46.5 (95% CI 43.5–50.1) and 59.8 (48.8–73.9) months for CLM and RLM patients, respectively. For CLM the median RFS and OS was 10.2 (95% CI 5.8–14.4) and 34.4 (30.1–42.1) months, respectively. In case of RLM the median RFS and OS were 8.6 (6.6–12.4) and 41.1 (35.2–48.6) months, respectively.

Primarily, the prognostic role of different parameters for RFS was tested. The parameters with significant effect (*p* < 0.05) or close to the significance level (*p* < 0.1) in univariate analysis or significant (*p* < 0.05) in multivariate analysis were included in Tables 3 and 4. To avoid multicollinearity some parameters had to be omitted, therefore NLR and SII were tested in two different multivariate model (Cox1 and Cox2). Both, NLR and SII proved to be independent markers of RFS in case of RLM, while only NLR was independent marker of RFS of CLM (Tables 3, 4).

**TABLE 3:** Uni- and multivariate analysis of RFS and OS for CLM.

Parameters	mRFS (95%CI)	p	HR_Cox1_ (95%CI)	p_Cox1_	HR_Cox2_ (95%CI)	p_Cox2_
Age						
<62	6.6 (4.4–8.9)	7 × 10^−4^	-		-	
≥62	14.6 (10.2–16.8)					
Resection margin						
R0	13.8 (8.3–17.8)	0.001	1 (ref)	0.048	1 (ref)	0.182
R1	6.6 (5.2–10)		1.42 (1.003–2.1)		1.3 (0.89–1.9)	
Synchronous						
yes	8.2 (2–8.2)	0.012	-		-	
no	12.9 (7–14.6)					
Postoperative chemotherapy ± targeted therapy						
none	5.5 (2–5.8)	3 × 10^−4^	-		1 (ref)	0.012
yes	13.4 (9.9–16.3)				0.61 (0.42–0.9)	
Pre- or postoperative chemotherapy ± targeted therapy						
none	2.5 (2–2.5)	0.006	-		-	
yes	10.8 (8.2–13.9)					
WBC						
<6.8	14.6 (10.8–19.3)	0.011	-		-	
≥6.8	8.3 (5.5–10.2)					
Neutrophil						
<4	19.3 (10.8–24.9)	0.002	-		-	
≥4	8.3 (5.8–10.5)					
NLR						
<2	14.6 (6.8–24.2)	0.004	1 (ref)	0.03	-	
≥2	9.9 (6–12.7)		1.44 (1.04–1.99)			
SII						
<535	14.4 (10.5–19.3)	0.005	-		1 (ref)	0.229
≥535	8.2 (5–9.9)				1.24 (0.87–1.78)	
GOT						
<24	12.9 (8.3–17.8)	0.043	1 (ref)	0.043	1 (ref)	0.013
≥24	6.8 (5.5–10.5)		1.43 (1.01–2.02)		1.6 (1.1–2.33)	
GPT						
<31	12.7 (7–14.4)	0.03	-		-	
≥31	5 (3.2–8.9)					
	**mOS (95% CI)**					
WBC						
<7.1	NR (40.5–46.6)	0.004	-		-	
≥7.1	30.1 (23.2–37.5)					
Neutrophil						
<4.8	NR (34.4–46.6)	0.004	-		1 (ref)	0.114
≥4.8	30.1 (23.2–37.5)				1.45 (0.91–2.31)	
Lymphocyte						
<2	68.3 (42.1–68.3)	0.005	-		1 (ref)	0.053
≥2	31.3 (24.2–34.4)				1.63 (0.99–2.67)	
Platelet						
<184	24.2 (17.9–33.1)	0.045	1 (ref)	0.853	-	
≥184	42.1 (31.3–68.3)		1.06 (0.59–1.9)			
GOT						
<24	NR (39.7–42.1)	0.021	1 (ref)	0.073	1 (ref)	0.233
≥24	31.3 (24.6–40.8)		1.64 (0.96–2.81)		1.4 (0.81–2.41)	
GPT						
<17	NR (−33.1)	0.011	-		-	
≥17	34.4 (25–40.8)					
Extent of progression						
single	37.5 (31.1–46.6)	0.016	1 (ref)	0.043	1 (ref)	0.011
multiple	20.3 (16.3–24.2)		1.71 (1.02–2.88)		1.98 (1.17–3.34)	

CI, confidence interval; Cox(1 or 2), multivariate Cox regression analysis (model 1 or 2); GOT, aspartate aminotransferase; GPT, alanine aminotransferase; HR, hazard ratio; mOS, median overall survival; mRFS, median relapse-free survival; NLR, neutrophil-to lymphocyte ratio; NR, not reached; ns, not significant; ref, reference; SII, systemic immune-inflammation index; WBC, white blood cells.

**TABLE 4:** Uni- and multivariate analysis of RFS and OS for RLM.

Parameters	mRFS (95%CI)	p	HR_Cox1_ (95%CI)	p_Cox1_	HR_Cox2_ (95%CI)	p_Cox2_
Resection margin						
R0	11.7 (7.1–17.3)	0.005	1 (ref)	0.001	-	
R1	6.5 (4.8–11.1)		1.58 (1.2–2.07)			
Postoperative chemo ± targeted therapy						
none	6.6 (4.1–8.6)	0.084	1 (ref)	0.014	1 (ref)	0.114
yes	11.1 (7–15.4)		0.69 (0.52–0.93)		0.79 (0.6–1.06)	
WBC						
<7.3	10.9 (6.5–15.6)	0.06	1 (ref)	0.568	1 (ref)	0.416
≥7.3	6.9 (4.8–11.5)		1.09 (0.81–1.48)		1.13 (0.84–1.52)	
Neutrophil						
<5.5	11.1 (7.1–15.6)	0.014	-		-	
≥5.5	6.6 (4.2–8.3)					
Lymphocyte						
<0.97	13.6 (6.6–23.7)	0.025	-		-	
≥0.97	7.1 (6.2–11.2)					
NLR						
<2.42	6.3 (4.8–7.6)	1.6 × 10^−4^	1 (ref)	0.021	-	
≥2.42	14.9 (7.8–19.2)		0.71 (0.54–0.95)			
SII						
<792	6.5 (5.7–7.6)	1.8 × 10^−4^	-		1 (ref)	0.002
≥792	19.2 (11.9–23.7)				0.65 (0.5–0.85)	
GPT						
<22	8.6 (5.9–14.2)	0.085	1 (ref)	0.151	1 (ref)	0.139
≥22	11.2 (6.2–24.2)		0.82 (0.63–1.07)		0.82 (0.63–1.07)	
	**mOS (95% CI)**					
Age						
<64	47.3 (40.2–62.4)	0.054	1 (ref)	0.004	1 (ref)	0.012
≥64	33.1 (26.1–39.2)		1.57 (1.15–2.14)		1.47 (1.09–1.99)	
Resection margin						
R0	52.5 (37.8–71.4)	0.002	1 (ref)	0.025	-	
R1	31 (20.9–41.1)		1.4 (1.04–1.88)			
Postoperative targeted therapy						
none	46.5 (36.8–62)	0.059	-		-	
yes	31.7 (20.3–40.5)					
WBC						
<4.2	31.2 (20.3–35.2)	0.036	1 (ref)	0.031	1 (ref)	0.005
≥4.2	46 (39.2–62.4)		0.67 (0.47–0.96)		0.58 (0.40–0.85)	
Lymphocyte						
<1.7	39.3 (31.7–46)	0.059	-		-	
≥1.7	101 (39.2–101)					
Platelet						
<314	39.2 (31.7–46)	0.032	1 (ref)	0.044	-	
≥314	NR (52.5–62.4)		0.47 (0.22–0.98)			
NLR						
<2.56	39.2 (26.7–41.1)	0.084	1 (ref)	0.038	-	
>2.56	47.3 (35.2–63.1)		0.72 (0.53–0.98)			
SII						
<742	36.8 (26.7–41.1)	0.011	-		1 (ref)	0.001
≥742	62 (41.2–138)				0.57 (0.41–0.79)	
GPT						
<13	26.5 (16.6–41.2)	0.237	1 (ref)	0.002	1 (ref)	0.006
≥13	40.2 (33.8–50.3)		0.54 (0.34–0.8)		0.58 (0.39–0.86)	

CI, confidence interval; Cox(1 or 2), multivariate Cox regression analysis (model 1 or 2); GPT, alanine aminotransferase; HR, hazard ratio; mOS, median overall survival; mRFS, median relapse-free survival; NLR, neutrophil-to lymphocyte ratio; NR, not reached; ns, not significant; ref, reference; SII, systemic immune-inflammation index; WBC, white blood cells.

**FIGURE 1:**
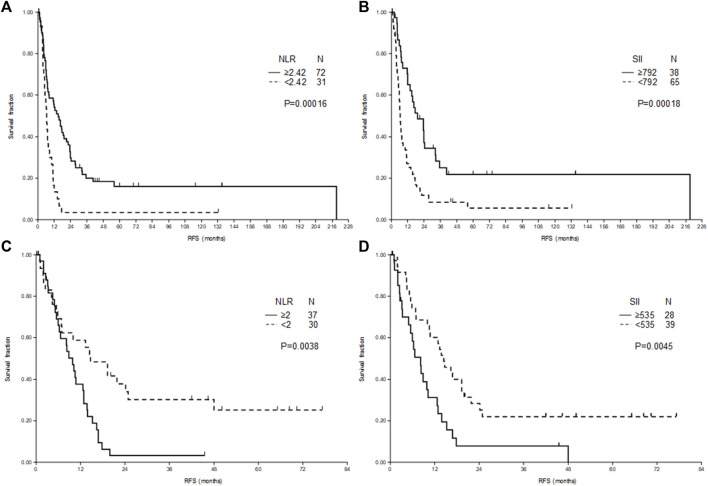
Relapse-free survival (RFS) after liver metastasectomy of patients with rectal cancer **(A,B)** and colon cancer **(C,D)** according to neutrophil-to-lymphocyte ratio (NLR) **(A,C)** and systemic immune-inflammation index (SII) **(B,D)**.

At 18 months only one patient (3%) remained free of relapse in the low NLR group of RLM, while in the high NLR group 28 patients (39%) were free of progression (Figure 1A). In contrast, at 18 months in low NLR group of CLM 50% of patients were free of relapse, while in the high NLR group all, but three patients progressed (Figure 1C). The number of patients free from relapse at 18 months for low and high SII of RLM was 10 (15%) and 19 (50%), respectively (Figure 1B) and for CLM was 15 (38%) and 3 (11%), respectively (Figure 1D).

Survival analysis of OS was conducted with the recalculated cut-off values for continuous variables. Besides, age, resection margin, GPT, platelet and WBC count, both NLR or SII proved to be significant predictors of OS in RLM (Table 4). In univariate analysis for CLM nor NLR neither SII was statistically significant predictor of OS (Table 3).

**FIGURE 2:**
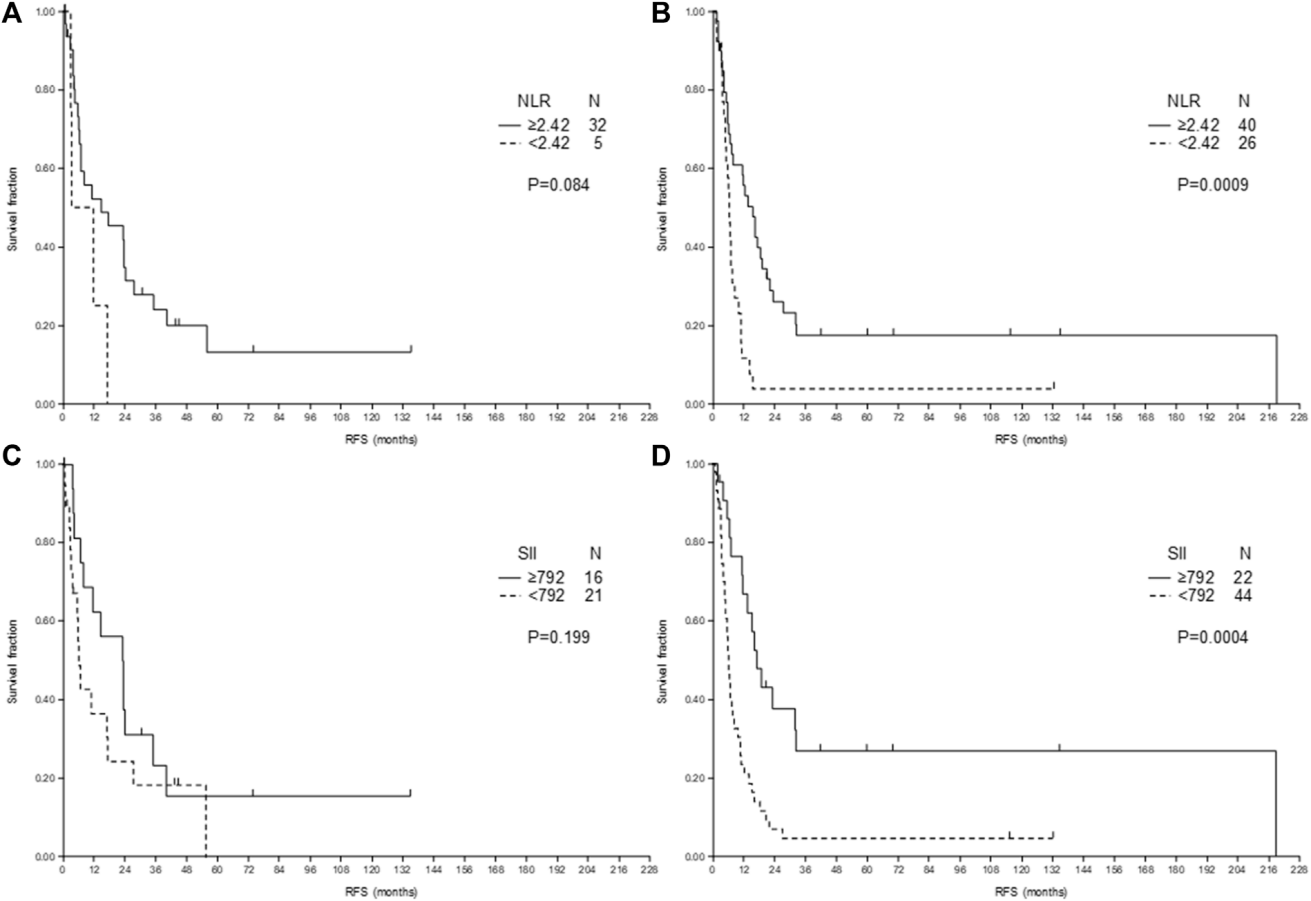
Relapse-free survival (RFS) after liver metastasectomy of patients with rectal cancer according to neutrophil-to-lymphocyte ratio (NLR) **(A,B)** and systemic immune-inflammation index (SII) **(C,D)** and according to treatment: no treatment **(A,C)** or chemotherapy (±targeted treatment) before metastasectomy **(B,D)**.

As Hand et al. [17] reported that preoperative chemotherapy influenced the role of NLR in CRLM, we analyzed the RFS stratified according to treatment before metastasectomy. In case of RLM the longer RFS of high NLR (Figures 2A,B) or SII (Figures 2C,D) was present both for treated and untreated patients, however, in case of untreated patients the significance level was not reached because of relatively low number of cases (Figure 2).

Preoperative treatment also did not influence the effect of NLR and SII in case of CLM (data not shown).

Moreover, if the preoperative treatment was included as covariate in multivariate tests of PFS and OS its effect was non-significant. A very similar result was found for SII, and the presence or not of targeted treatment prior metastasectomy also did not influence the results for NLR or SII (data not shown).

## Discussion

There are some articles which analyzed the role of NLR in prediction of RFS after resection of liver metastases of CRC patients. In some of them the localization of primary tumor was not reported or rectal tumors were studied together with the left sided colon tumors (Table 5).

**TABLE 5:** Literature data about RFS according to primary colorectal cancer (CRC) localization after metastasectomy of liver-only metastases.

Primary tumor localization	Ref no	N	Colon	Longer RFS	*p*	*p*	Preop	Sync	Postop	Follow-up^a^ months
			%	NLR (SII) cut-off	Univ	Multiv	%	%	%	
CRC										
	3, 26	586	NA	>5	0.272	-	23	38	27	>6
	6	440	NA	<5	<0.001	<0.001	11	33	>33	24
	8	247	NA	<5	0.77	-	-	-	-	20
	13	169	NA	<2.5	0.09	0.347	100	72	76	34.6
	14	140	NA	<2.4	0.033	0.609	100	71	74	33
	16	92	NA	<5	0.047	0.022	76	0	100	27.1^b^
COLON cancer										
	25	98	100	<2.5	0.044	0.029	-	0	-	35.2^b^
	Our study	67	100	<2	0.004	0.03	76	10	73	46.5
				(<535)	0.005	0.229				
>50% of patients with COLON cancer										
	9	575	78	≤5	0.104	-	86	66	90	37
	11	452	56	<2.6	0.163	-	63	53	74	28
				(≤517)	0.068	-				
	4	343	58	<2.5	0.017	-	58	49	-	49
	5	295	60	<2	<0.001?	0.001	19	78	37	63.2
	12	283	66	(≤0.0135)	0.003	0.005	51	58	77	35.4
	31	231	67	≤3	0.049	0.06	76	35	72	73.2
	30	197	62	<3	0.284	0.617	26	57	-	-
	15	183	57	<2.3	ns	-	100	85	78	36.3
	29	150	58	<4.63	0.017	0.452	39	63	73	36
	28	182	70	>3	0.939	-	19	65	91	32.5^b^
	27	128	79^c^	<1.71	ns	-	74	63	-	45
	32	130	54	≤5	0.044	0.03	16	38	26	44^b^
>50% of patients with RECTAL cancer										
	17	83	24	<1.94	0.026	0.006	29	100	100	-
	7, 33	174	46	<5	0.008	ns	42	0	39	36
RECTAL cancer										
	Our study	103	0	≥ 2.42	<0.001	<0.001	64	10	72	59.8
				(≥ 792)	<0.001	0.002				

CRC, colorectal cancer; multiv, multivariate; NA, not available; ns, non-significant; Postop, postoperative treatment; Preop, preoperative treatment; Ref, reference; RFS, relapse-free survival; SII, systemic immune-inflammation index; Sync, synchronous; univ, univariate.

a Median.

b Average.

c Personal communication.

In the few articles where the colon and rectum appears among patients’ characteristics, no stratified test was performed based on localization. There is only one article where exclusively colon tumors were analyzed. To the best of our knowledge, the present study is the first investigation, which performed a separate analysis of rectal tumors. All relevant studies have been summarized in Table 5.

Since no study has been found where the high NLR would be a significant marker of longer RFS, in case of CLM the association between high NLR and shorter RFS is obvious. For RLM there are very few articles where patients with primary rectal cancer were in the majority. [7, 33] examined the role of NLR in two articles (88% overlap with patient data), and found longer RFS and OS for low NLR, however their result was in correlation with the high frequency of postoperative infectious complications (77% of all postoperative complications) [33], which was lower in our study (29%, data not shown). Kim et al. [17] reported similar result, however, their study investigated only patients with synchronous surgery of primary and metastases and all patients received adjuvant chemotherapy. Moreover, there were far fewer preoperatively-treated patients in Kim’s article (29%) than in ours (64%) or in Neal’s reports (42%). Can the preoperative treatment alter the results? Interestingly, in Table 3 of the six reports where colon cancer patients were in the majority, in five where preoperative treatment was common (>60%), NLR was not significant for RFS. On the other hand, in four out of six reports with lower preoperative treatment frequency (<60%) the NLR was significant marker of RFS. Hand et al. [18] reported NLR as a non-significant or significant marker of OS (RFS was not studied) after liver metastasectomy of CRC patients (the location of primary tumor was not reported) depending whether the patients received or not preoperative chemotherapy, respectively. In our study, in contrast to the results of [18] in patients who received preoperative treatment the high NLR proved to be a very significant predictor of longer RFS. The discrepancy can be explained by different preoperative treatment (chemotherapy only in Hand’s study vs chemotherapy+targeted therapy in >50% of patients in our study) and different location of the primary tumor, as we have seen that in case of rectum and colon, the role of NLR is completely different. In spite that the influence of chemo- and targeted therapies on NLR of patients with advanced CRC [34] and on immunologic characteristics of liver metastases of CRC tumors [35] was already reported, further studies should clarify the effect of preoperative treatment on NLR in case of liver metastases of colon and separately rectal cancer.

A publication studying SII in CRLM patients was reported by [11], but neither NLR (Table 3), nor SII was significant for RFS after metastasectomy. Another recent study by [12] found significantly longer survival for low SII, which proved to be independent predictor of RFS. Their results may differ from that ours because they investigated colon (56–66%) and rectal cancer patients together (Table 3).

Our results can also be an explanation why [4] concluded that an integrated cut-off value can’t be determined for the preoperatively measured NLR in CRLM patients. The various colon/rectum ratio and different frequencies of preoperative treatment in reviewed studies made the results inconsistent. Therefore, it is important to primarily shed light on the difference between colon and rectum, and not to find a cut-off that could be used in the clinic.

The histologic, genetic, behavioral, etc. differences between colon and rectum tumors detailed by [22] and moreover the inflammation pattern (IL-6, CRP), which differs in colon and rectum tumors described by [23, 24] may explain our results.

McCoy et al. [36] studied the relation between the presence of stromal Foxp3 and RFS in rectal cancer patients after preoperative treatment and reported a significantly longer RFS (*p* = 0.025) for low Foxp3+ cell density. In another study, [37] demonstrated a strong negative correlation (*p* = 0.006) between stromal Foxp3+ infiltration and preoperative serum CRP levels in CRC patients. A study by [38] showed that high preoperative CRP levels were associated (*p* < 0.001) with high NLR in CRC patients. According to the above data it can be hypothesized that RLM patients with high NLR, which is associated with high CRP and subsequently with low Foxp3 levels may have longer RFS, as a consequence of a specific tumor immune microenvironment (TIME).

The longer RFS for colon in cases of low NLR may be explained by the presence of tumor-infiltrating lymphocytes (TILs). According to the results of [39] the low NLR in CRC patients (73% colon cancer) was significantly associated with higher TILs (*p* = 0.005) at the invasive margin of the tumor and a significantly longer DFS. CD8^+^ TILs may account for antitumoral effect, resulting in an unfavorable tumor immune microenvironment (TIME) and longer RFS. [40] studied 94 liver metastases of CRCs (65% colon cancer) and the level of CD8^+^ TILs. The distribution of high and low CD8^+^ density was significantly different for rectal and colon origin. A high CD8^+^ was more frequently observed for colon (54%) than for rectal origin (30%, *p* = 0.027). There was no difference between left and right sided colon. The RFS was significantly better (*p* = 0.018) for cases with high CD8^+^. Similarly, [41] reported a similar result, that high CD8/CD3 ratio was significantly more frequent in intra- and peritumoral tissue of liver metastases of colon tumors (60% and 54%) than that of rectal tumors (37% and 35%, *p* = 0.011 and 0.035, respectively). The RFS for high CD8/CD3 was significantly longer (*p* = 0.035). Other results of studies investigating NLR on recurrence of primary colon or rectal tumors can be used for comparison only with reservations, because the CRLM differ from primary lesions in terms of immune cell infiltration [42, 43, 44].

In accordance with [45] the location of primary tumor did not influence the RFS after hepatic resection. Instead, NLR, which reflects TIME, can influence the survival in different manner depending on primary localization.

The limitation of this study consist in its retrospective character. The treatments administered after relapse (surgery, chemotherapy, etc) were not considered for OS analysis. The prognostic factor (RAS mutation) [2, 8] and other tumor markers (e.g. fibrinogen/albumin index [11] or prognostic nutrition index [5], etc.) were also not available. In spite of limitations, this study has the power to clarify the controversies, which still exists between NLR and RFS after liver resection of patients with CRLM.

### Conclusion

The NLR and SII determined before surgery of liver metastases of patients with RLM proved to be an independent prognostic factor of RFS and OS in an opposite manner as for CLM where low NLR predicts longer RFS, namely NLR and SII above the cut-off level predicts longer RFS. Further prospective studies stratified according to primary tumor location and TIME may strengthen our findings.

## Data Availability

The datasets presented in this article are not readily available due to patient privacy. Requests to access the datasets should be directed to the corresponding author.
