# Corrigendum: High Neutrophil-to-Lymphocyte Ratio (NLR) and Systemic Immune-Inflammation Index (SII) are Markers of Longer Survival After Metastasectomy of Patients With Liver-Only Metastasis of Rectal Cancer

**DOI:** 10.3389/pore.2022.1610658

**Published:** 2022-10-06

**Authors:** Nándor Polk, Barna Budai, Erika Hitre, Attila Patócs, Tamás Mersich

**Affiliations:** ^1^ Department of Laboratory Medicine, Semmelweis University, Budapest, Hungary; ^2^ Departmet of Visceral Surgery, National Institute of Oncology, Budapest, Hungary; ^3^ Department of Molecular Genetics, National Institute of Oncology, Budapest, Hungary; ^4^ Medical Oncology and Clinical Pharmacology “B” Department, National Institute of Oncology, Budapest, Hungary; ^5^ Clinical Central Laboratory, National Institute of Oncology, Budapest, Hungary

**Keywords:** neutrophil-to-lymphocyte ratio, liver-only metastases of rectal cancer, metastasectomy, relapse-free survival, systemic immune-inflammation index, liver-only metastases of colon cancer, preoperative treatment

In the published article, there was an error in the order of the affiliations. The original list read:

Nándor Polk^1^, Barna Budai^2,^*, Erika Hitre^3^, Attila Patócs^2,4,5^ and Tamás Mersich^1^

^1^Departmet of Visceral Surgery, National Institute of Oncology, Budapest, Hungary

^2^Department of Molecular Genetics, National Institute of Oncology, Budapest, Hungary

^3^Medical Oncology and Clinical Pharmacology “B” Department, National Institute of Oncology, Budapest, Hungary

^4^Clinical Central Laboratory, National Institute of Oncology, Budapest, Hungary

^5^Department of Laboratory Medicine, Semmelweis University, Budapest, Hungary

The corrected list is:

Nándor Polk^2^, Barna Budai^3,^*, Erika Hitre^4^, Attila Patócs^1,3,5,^* and Tamás Mersich^2^

^1^Department of Laboratory Medicine, Semmelweis University, Budapest, Hungary

^2^Departmet of Visceral Surgery, National Institute of Oncology, Budapest, Hungary

^3^Department of Molecular Genetics, National Institute of Oncology, Budapest, Hungary

^4^Medical Oncology and Clinical Pharmacology “B” Department, National Institute of Oncology, Budapest, Hungary

^5^Clinical Central Laboratory, National Institute of Oncology, Budapest, Hungary

A new corresponding author has been included. The correct correspondence footnote is:

*Correspondence: Barna Budai, budai.barna@oncol.hu; Attila Patócs, patocs.attila@med.semmelweis-univ.hu

The authors apologize for these errors and state that this does not change the scientific conclusions of the article in any way. The original article has been updated.

